# Coronary Plaque Morphology and the Anti-Inflammatory Impact of Atorvastatin: A Multicenter ^18^F-Fluorodeoxyglucose Positron Emission Tomographic/Computed Tomographic Study

**DOI:** 10.1161/CIRCIMAGING.115.004195

**Published:** 2016-12-12

**Authors:** Parmanand Singh, Hamed Emami, Sharath Subramanian, Pal Maurovich-Horvat, Gergana Marincheva-Savcheva, Hector M. Medina, Amr Abdelbaky, Achilles Alon, Sudha S. Shankar, James H.F. Rudd, Zahi A. Fayad, Udo Hoffmann, Ahmed Tawakol

**Affiliations:** From the Division of Cardiology, New York Presbyterian Hospital and Weill Cornell Medical College (P.S.); Cardiac MR PET CT Program, Division of Cardiac Imaging (H.E., S.S., P.M.-H., G.M.-S., Amr Abdelbaky, U.H., A.T.) and Division of Cardiology (A.T.), Massachusetts General Hospital and Harvard Medical School, Boston; MTA-SE Cardiovascular Imaging Research Group, Semmelweis University, Budapest, Hungary (P.M.-H.); Fundacion Cardio-Infantil, Bogota, Colombia (H.M.M.); Merck and Company, Inc, Kenilworth, NJ (Achilles Alon, S.S.S.); Division of Cardiovascular Medicine, University of Cambridge, United Kingdom (J.H.F.R.); and Translational and Molecular Imaging Institute, Icahn School of Medicine at Mount Sinai, New York, NY (Z.A.F.).

**Keywords:** atherosclerosis, carotid artery, coronary artery disease, inflammation, positron emission tomography

## Abstract

**Background—:**

Nonobstructive coronary plaques manifesting high-risk morphology (HRM) associate with an increased risk of adverse clinical cardiovascular events. We sought to test the hypothesis that statins have a greater anti-inflammatory effect within coronary plaques containing HRM.

**Methods and Results—:**

In this prospective multicenter study, 55 subjects with or at high risk for atherosclerosis underwent ^18^F-fluorodeoxyglucose positron emission tomographic/computed tomographic imaging at baseline and after 12 weeks of treatment with atorvastatin. Coronary arterial inflammation (^18^F-fluorodeoxyglucose uptake, expressed as target-to-background ratio) was assessed in the left main coronary artery (LMCA). While blinded to the PET findings, contrast-enhanced computed tomographic angiography was performed to characterize the presence of HRM (defined as noncalcified or partially calcified plaques) in the LMCA. Arterial inflammation (target-to-background ratio) was higher in LMCA segments with HRM than those without HRM (mean±SEM: 1.95±0.43 versus 1.67±0.32 for LMCA with versus without HRM, respectively; *P*=0.04). Moreover, atorvastatin treatment for 12 weeks reduced target-to-background ratio more in LMCA segments with HRM than those without HRM (12 week-baseline Δtarget-to-background ratio [95% confidence interval]: −0.18 [−0.35 to −0.004] versus 0.09 [−0.06 to 0.26]; *P*=0.02). Furthermore, this relationship between coronary plaque morphology and change in LMCA inflammatory activity remained significant after adjusting for baseline low-density lipoprotein and statin dose (β=−0.27; *P*=0.038).

**Conclusions—:**

In this first study to evaluate the impact of statins on coronary inflammation, we observed that the anti-inflammatory impact of statins is substantially greater within coronary plaques that contain HRM features. These findings suggest an additional mechanism by which statins disproportionately benefit individuals with more advanced atherosclerotic disease.

**Clinical Trial Registration—:**

URL: http://www.clinicaltrials.gov. Unique identifier: NCT00703261.

## Abstract 2

Supplemental Digital Content is available in the text.

It is well accepted that the presence of nonobstructive atherosclerotic plaques, particularly those with high-risk morphological (HRM) features, is associated with an increased risk of future cardiovascular disease events.^1,2^ This observation has led to proposals to use data on nonobstructive plaque characteristics (if available) to alter the thresholds for prescribing lipid-lowering drugs for primary prevention.^3,4^ A related and yet unresolved question is whether the antiatherosclerotic benefits of statins may be differentially imparted depending on the severity of the underlying atherosclerotic process. This question is of substantial importance because an association between atherosclerotic disease morphology and statin efficacy would provide a second reason to consider structural disease characteristics when deciding on primary prevention therapy.

**See Editorial by Mehta**

**See Clinical Perspective**

Atherosclerosis is a systemic inflammatory condition^5^; the anti-inflammatory actions of statins is one of its most important beneficial effects on atherosclerosis.^6^ Arterial imaging using 18-fluorodeoxy glucose positron emission tomography and computed tomography (FDG-PET/CT) is used to reproducibly measure atherosclerotic inflammation.^7–10^ Atherosclerotic FDG uptake can be quantified noninvasively and significantly correlates with systemic inflammatory biomarkers,^11^ macrophage infiltration, and inflammatory cell gene expression within the arterial wall.^12,13^ Furthermore, it provides an independent marker of risk for future atherothrombotic events^14,15^ and is modifiable by statin therapy.^9,16^ The majority of FDG-PET imaging studies have focused on extracoronary vessels.^7,10,17^ However, successful evaluation of coronary FDG uptake has been reported previously^18^ and has recently focused on the left main coronary artery (LMCA) segment, which does not have an epicardial course and thus lends itself to measurement of FDG uptake.^19^

We hypothesized that atherosclerotic inflammation is greatest within LMCA manifesting HRM features. Accordingly, in this prospective treatment study, we tested the hypothesis that the anti-inflammatory actions of atorvastatin are greatest in LMCA with (versus without) HRM.

## Methods

### Study Design

This study is part of a larger randomized double-blind trial that was conducted at 10 US centers to investigate the impact of atorvastatin therapy on arterial wall inflammation. The study complies with the Declaration of Helsinki. The protocol was reviewed and approved by each center’s institutional review board, and all participants provided written informed consent before any study procedures. Analysis of FDG uptake in the LMCA, carotid arteries, and aorta was performed before unblinding of the data. In the current study, we used computed tomographic angiography (CTA) images and performed a blinded analysis of plaque composition in carotids and coronary arteries and additionally assessed the relationship between FDG uptake and high-risk plaque morphology. Permission was received from the Partners Healthcare Institutional Review Board to perform these additional analyses on the anonymized images. The primary end point of the study, evaluating the effect of statin therapy on large-vessel inflammatory activity (assessed by FDG PET/CT), was previously reported.^9^

### Patients

One hundred and sixty-three subjects were initially screened, and 83 subjects (78% men; median age: 59 years; range: 37–78 years) underwent baseline FDG-PET/CT imaging. Criteria for inclusion were individuals aged 30 to 80 years with documentation or history of any one of the following: (1) coronary artery disease, (2) carotid artery disease, (3) cerebrovascular disease, (4) peripheral arterial disease (ankle–brachial index ≥0.5 and ≤0.9), (5) type 2 diabetes mellitus, and (6) body mass index 30 to 40 kg/m^2^ or waist circumference >102 cm in men and >88 cm in women. Subjects were excluded if they had a history of (1) type 1 diabetes mellitus, (2) significant cardiovascular event or intervention within 12 weeks of screening, (3) significant heart failure (ie, New York Heart Association class III or IV), (4) hepatobiliary disease, (5) chronic systemic inflammatory condition (such as rheumatoid arthritis or psoriasis), or (6) chronic infection. In addition, eligible subjects were required to have low-density lipoprotein cholesterol level ≥60 mg/dL and triglyceride level <350 mg/dL and were required to be statin naive or taking no more than low-dose statins (defined as atorvastatin ≤10 mg, simvastatin ≤20 mg, rosuvastatin ≤5 mg, pravastatin ≤40 mg, or fluvastatin ≤40 mg).

After the initial clinical screening, subjects underwent baseline imaging with FDG-PET/CT. Because the study was originally designed to evaluate the effect of statin treatment on arterial wall inflammation, subjects without evidence of arterial inflammation (ie, only subjects with target-to-background ratios [TBR] ≥1.6 in either the aorta or carotids were included) at baseline were excluded before randomization, resulting in the exclusion of ≈10% of the initially screened population. Follow-up FDG-PET/CT images were obtained after 12 weeks of atorvastatin therapy.

### FDG-PET/CT Imaging

FDG-PET/CT imaging was performed using previously reported approaches.^9^ Briefly,^18^F-FDG was administered intravenously (≈10 mCi for a 70 kg patient) after an overnight fast, and imaging was performed 90 minutes after FDG injection using a hybrid PET/CT scanner (Seimens Biograph 64 or similar). To suppress myocardial FDG uptake, subjects were instructed to consume a low-carbohydrate, high-fat diet starting with the dinner meal (or 5.00 pm, whichever comes first), the evening before the day of FDG-PET/CT imaging, to suppress myocardial FDG uptake. An attenuation correction CT scan using 140 kVp was performed followed by PET imaging of the neck and chest, with 15-minute acquisitions per bed position. The reconstruction of attenuation-corrected images was performed using ordered subset expectation maximization algorithm. All patients had a blood sugar concentration <200 mg/dL at the time of imaging.

### Measurement of Coronary Artery FDG Uptake

PET/CT images were analyzed using previously detailed methods.^18,19^ Investigators were blinded to the clinical history and temporal sequence of the images. Subsequently, the data sets (week 0 and 12 images) were batch analyzed after coregistration of PET and CT images (Leonardo TrueD; Siemens, Forchheim, Germany). Maximum standardized uptake value of FDG was measured in LMCA by placing 4 regions of interest at the LMCA ostium, 5 and 10 mm distal to the ostium, and LMCA bifurcation. The measurements from those LMCA locations were averaged to produce mean of maximum LMCA standardized uptake value. Care was taken to avoid the spillover activity from the myocardium, as performed in a previous study by our group.^18^ The fact that coronary artery activity measurements were limited to the LMCA, a nonepicardial segment of the coronary tree, facilitated the avoidance of myocardial activity. Target-to-background ratios were derived from the ratio of the arterial standardized uptake value to background venous blood standardized uptake value from the superior vena cava. In addition, FDG uptake was measured in the aorta and carotids using previously described methods.^9^

### Measurement of Arterial Inflammation in the Aorta and Carotids

FDG uptake (TBR) was evaluated in 3 extracoronary arterial locations (right and left carotid and aorta). The extracoronary artery with the highest FDG uptake at baseline was identified as the index vessel, as previously described.^9^ The mean TBR of the index vessel was defined as the average of the maximum TBR activity for all of the axial segments that compose the index vessel.

### CT Angiography

Coronary CTA, to assess coronary artery plaque morphology, was performed in helical mode on multidetector CT scanners with ≥64 slices. Imaging parameters included rotation time of ≤420 ms, tube current of ≈750 to 850 mAs (effective), and voltage of 120 kVp. Image acquisition characteristics were slice thicknesses of 0.75 mm and pitch of 0.2 to 0.4. Iopamidol 300 mg/mL or a similar drug was used as an intravenous contrast agent and infused at 5 to 6 mL/s. Immediately after coronary CT angiography, the table was repositioned for acquisition of carotid images. Settings for carotid CT scanning were as follows: pitch 2.8, rotation time of ≤500 ms, tube current of ≈180 to 300 mA, and voltage of 120 kVp.

### Assessment of Atherosclerotic Plaque Morphology in the Coronary Arteries

Evaluation of the CTA images was performed while blinded to PET data and clinical information. Plaque morphology was assessed in the LMCA. HRM plaques in the LMCA were identified by presence of noncalcified or partially calcified plaques as described in previous studies.^20,21^ An analysis examining morphological features of atherosclerotic plaques in the entire coronary arterial tree and carotid circulation was also performed (please refer to the Table I and Figure I in the Data Supplement, respectively).

### Assessment of Blood Biomarkers

C-reactive protein (CRP) and matrix metalloproteinase-3 (MMP-3) concentrations were obtained at baseline and 12 weeks. Serum biomarker analyses were performed in batches.

### Statistical Analysis

Descriptive data are presented as mean±SD for continuous normally distributed variables, median (interquartile range) for continuous non-normally distributed data, and frequency with proportions for nominal variables, as appropriate. Independent samples *t* test was used for cross-sectional comparison of normally distributed continuous variables. Mann–Whitney *U* test was used for the similar analyses of continuous variables without normal distribution (such as CRP). A linear regression model was fitted to adjust for potential confounding factors on the effects of statin therapy in reduction of FDG uptake (TBR). The potential confounders for adjustment were selected based on the previous studies or clinical relevance. In the linear regression model to adjust for baseline left main TBR, we used tertiles of baseline TBR. Adjustment for Fisher exact test was performed for comparison of dichotomous variables. Pearson correlation coefficient (R) was used to assess correlations between continuous variables once normal distribution was verified, and Spearman ρ was reported as correlation coefficient for non-normally distributed variables. Two-tailed probability values are reported, and statistical significance is defined as *P*<0.05. All statistical analyses were performed using SPSS version 22 (IBM, Armonk, NY).

## Results

**Figure 1.**
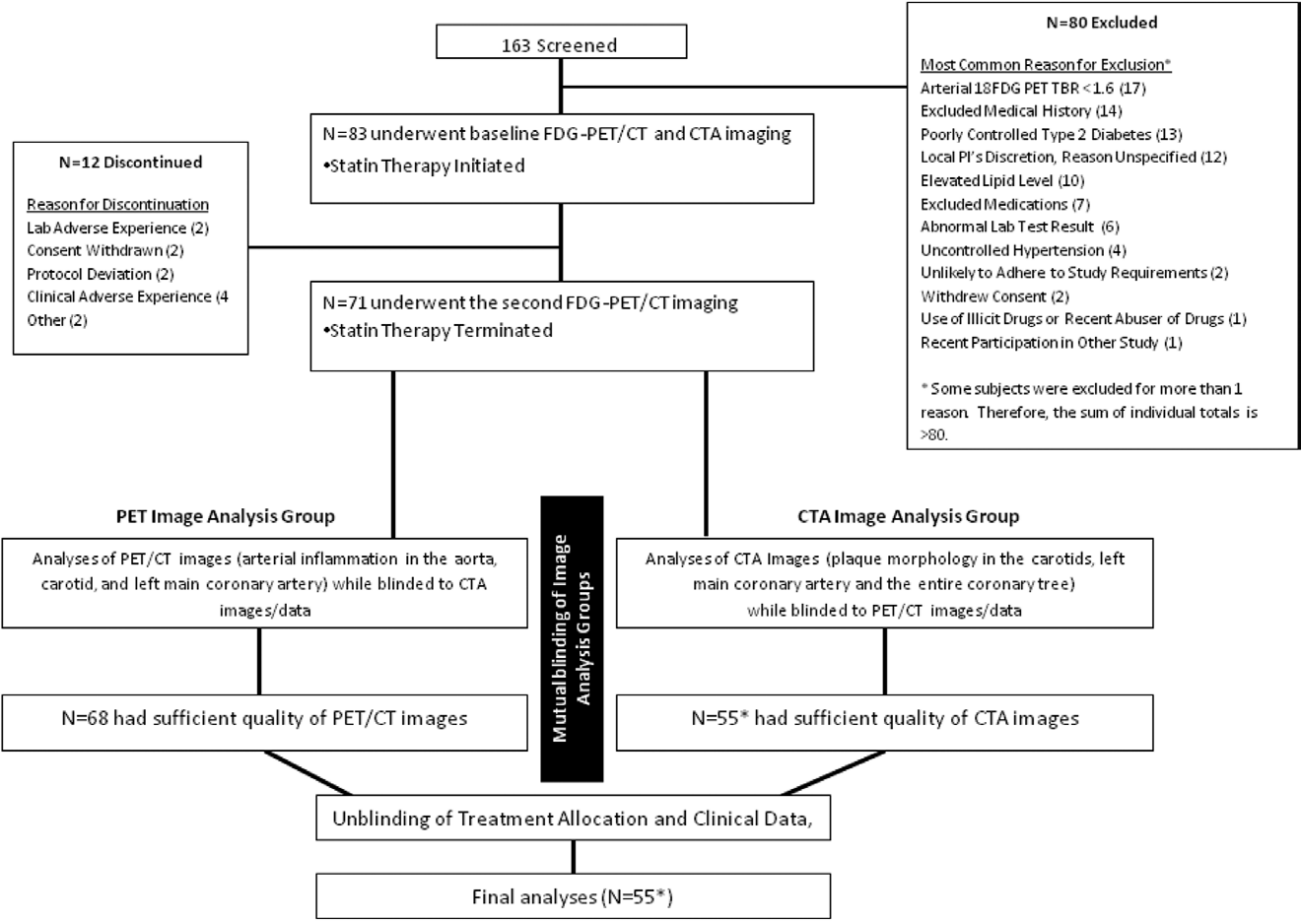
Study flow. Out of 163 subjects who were initially screened, 83 subjects underwent baseline ^18^F-flurodeoxyglucose positron emission tomographic (FDG-PET)/computed tomographic (CT) scan followed by statin treatment with atorvastatin. Follow-up FDG-PET scan was performed after 12-wk statin therapy. Seventy-one subjects completed the study, and 68 had evaluable PET/CT images. Thereafter, an independent reader analyzed 55 evaluable coronary and carotid computed tomographic angiography (CTA) images while blinded to PET data.

Baseline characteristics of study subjects are detailed in the Table. Seventy-one subjects completed the 12-week treatment period. In 68 of those subjects, PET/CT image quality was sufficient to analyze FDG uptake in the LMCA and extracoronary vessels (ie, aorta and carotids). Fifty-five subjects provided adequate-quality coronary CTA images to assess plaque morphology in the LMCA and carotid arteries (Figure 1).

**Table.**
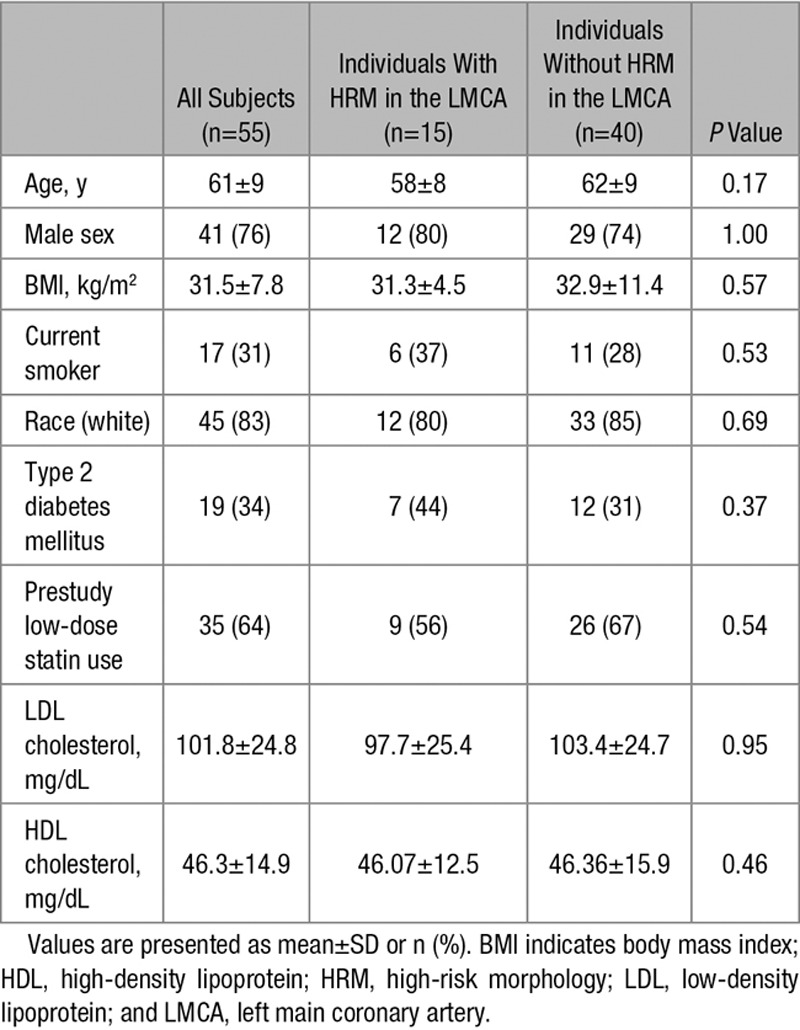
Baseline Characteristics of Study Subjects

### Left Main Coronary Arteries Containing High-Risk Plaque Morphological Features Have Greater Arterial Wall Inflammation

**Figure 2.**
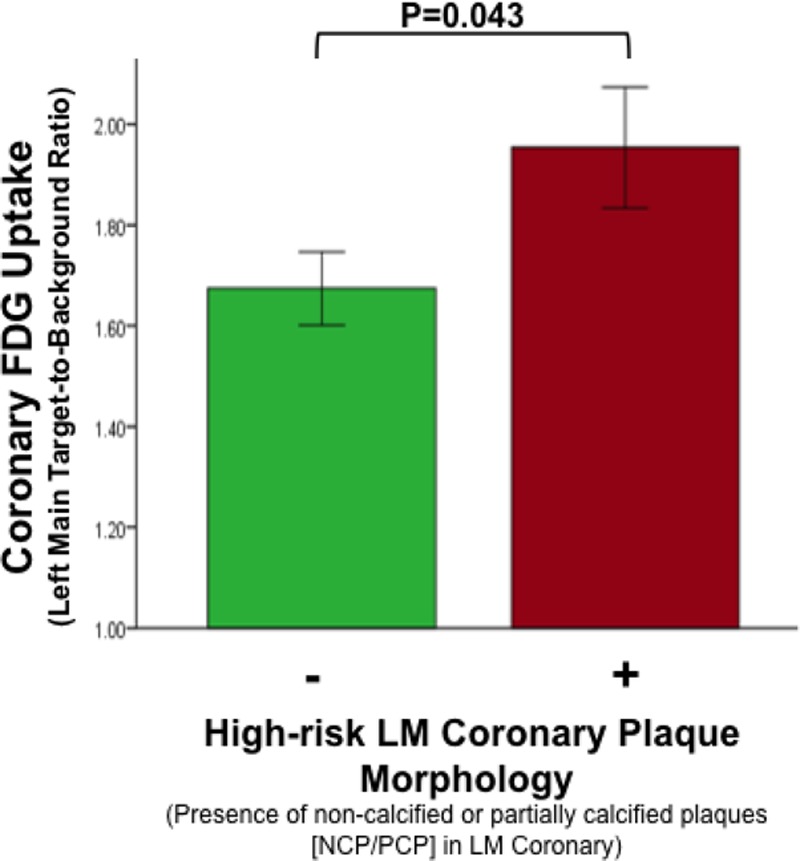
Association between high-risk plaque morphology and ^18^F-flurodeoxyglucose (FDG) uptake in left main coronary artery (LMCA). LMCA inflammation (target-to-background ratio [TBR]) in the index vessel was significantly higher in subjects with high-risk morphology (HRM) than those without HRM (partially calcified plaque/noncalcified plaque [NCP/PCP]) in the underlying coronary segment as detected by coronary computed tomographic angiography. Error bars represent SEM.

**Figure 3.**
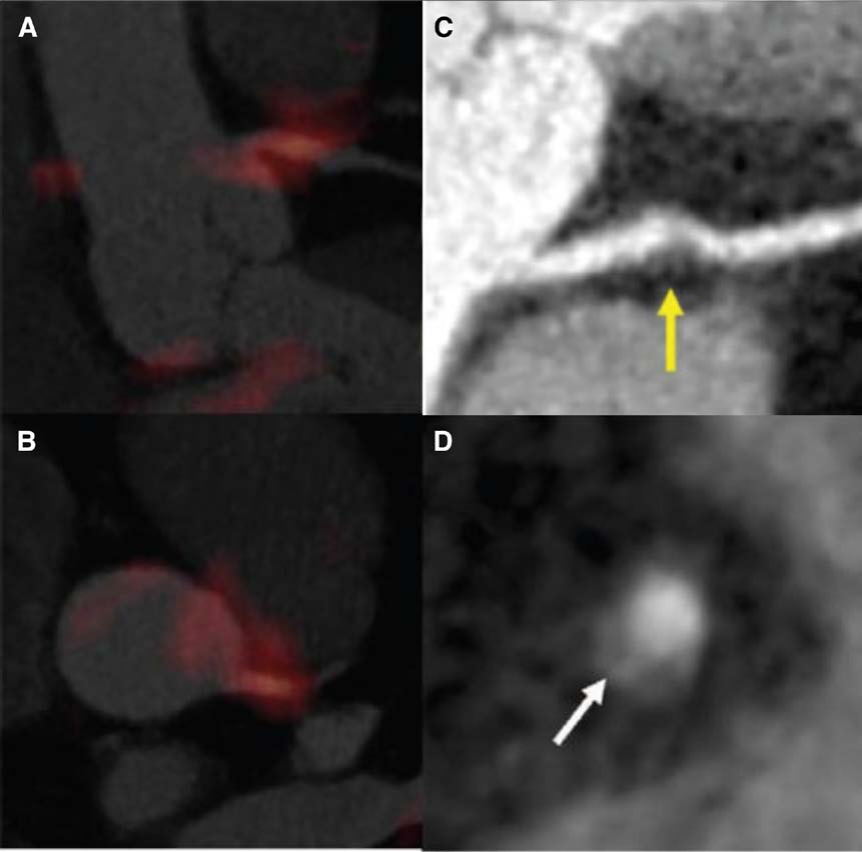
Focal ^18^F-flurodeoxyglucose (FDG) uptake in patients with high-risk plaque morphology in the left main coronary artery (LMCA). Fused positron emission tomographic (PET)/computed tomographic (CT) image showing intense and focal FDG uptake in the LMCA, in orthogonal images (**A** and **B**), corresponding maximum intensity projection–reconstructed computed tomography angiographic (CTA) image of LMCA with noncalcified plaque (arrow; **C**), and axial CTA showing a cross-sectional view (**D**) of an additional plaque in the right coronary artery manifesting positive remodeling and low attenuation (arrow) in the same subject.

LMCA inflammation (TBR) was higher in LMCA with HRM (defined as noncalcified or partially calcified plaque) than those without HRM ([mean±SE]: 1.95±0.43 versus 1.67±0.32; *P*=0.043; Figures 2 and 3). A similar relationship between plaque morphology (as determined by CTA) and arterial inflammation (as determined by FDG-PET/CT) was seen in the carotid arteries (see Figure I in the Data Supplement).

### Statins Reduce Left Main Coronary Artery Inflammation, an Effect More Pronounced Within Advanced Coronary Lesions

**Figure 4.**
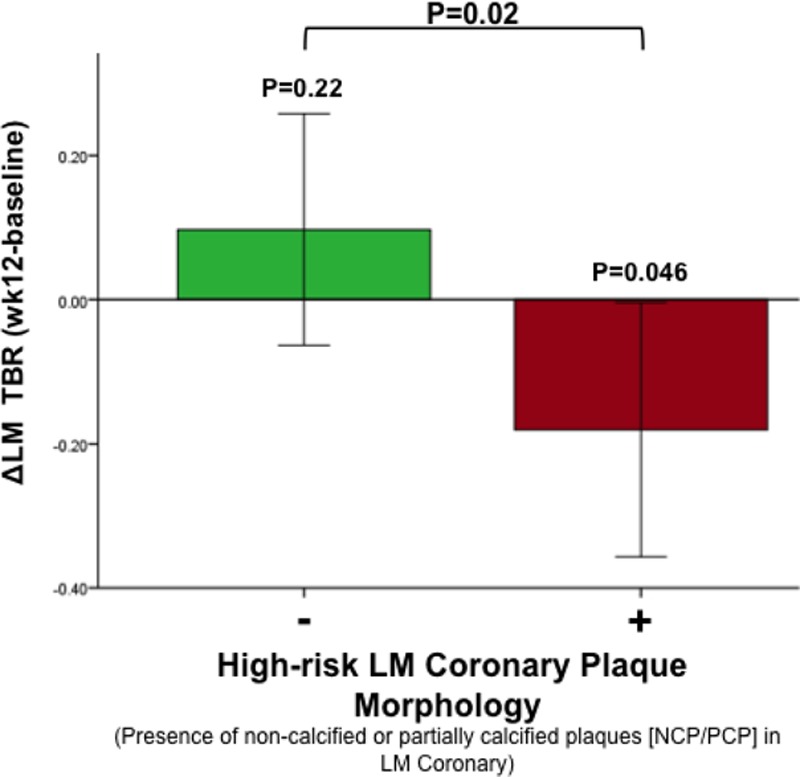
Statin therapy results in a greater reduction of ^18^F-flurodeoxyglucose (FDG) uptake in left main coronary artery (LMCA) with high-risk morphology (HRM). Changes in LMCA target-to-background ratio after 12-wk statin therapy were more pronounced in arteries with HRM in coronary computed tomographic angiography. Error bars represent SEM. NCP indicates noncalcified plaque; and PCP, partially calcified plaque.

We evaluated the impact of statin therapy on LMCA and observed that after 12 weeks of statin therapy, FDG uptake was reduced in LMCA with HRM but not in LMCA without HRM (12-week baseline change in TBR [95% confidence interval]: −0.18 [−0.35 to −0.004] versus 0.09 [−0.06 to 0.26]; *P*=0.02; Figure 4). This relationship remained significant in a multivariate model after adjusting for prestudy statin use and statin dose after randomization (β=−0.286; *P*=0.02). The relationship remained statistically significant after adjusting for baseline TBR (β=−0.26; *P*=0.04). Additionally, the relationship between LMCA plaque morphology and change in LMCA inflammation (TBR) after atorvastatin remained significant after adjusting for body mass index (β=−0.27; *P*=0.02) and after adjusting for baseline low-density lipoprotein and statin dose (β=−0.27; *P*=0.038).

### Association Between Imaging Measures and Serum Biomarkers

For the comparison between blood biomarkers and coronary plaques, we assessed plaque morphology across the entire coronary tree (see Data Supplement). Because of the relative abundance of plaques across the entire coronary tree, we were able to use a more stringent definition of HRM, in this case, plaques with both positive remodeling and low attenuation (double positives). At baseline, CRP was significantly higher in subjects with HRM than those without HRM within the entire coronary arterial tree (median [interquartile range]=3.6 [1.0–4.8] mg/L versus 1.3 [0.6–2.5] mg/L; *P*=0.01). We did not observe significant association between CRP concentrations and LMCA inflammation (TBR), although we observed a correlation between serum MMP-3 and LMCA inflammation (TBR; ρ=0.31; *P*=0.04; *R*=0.42; *P*=0.004).

### Left Main Coronary Artery Inflammation Correlates With Inflammation in Extracoronary Arteries

**Figure 5.**
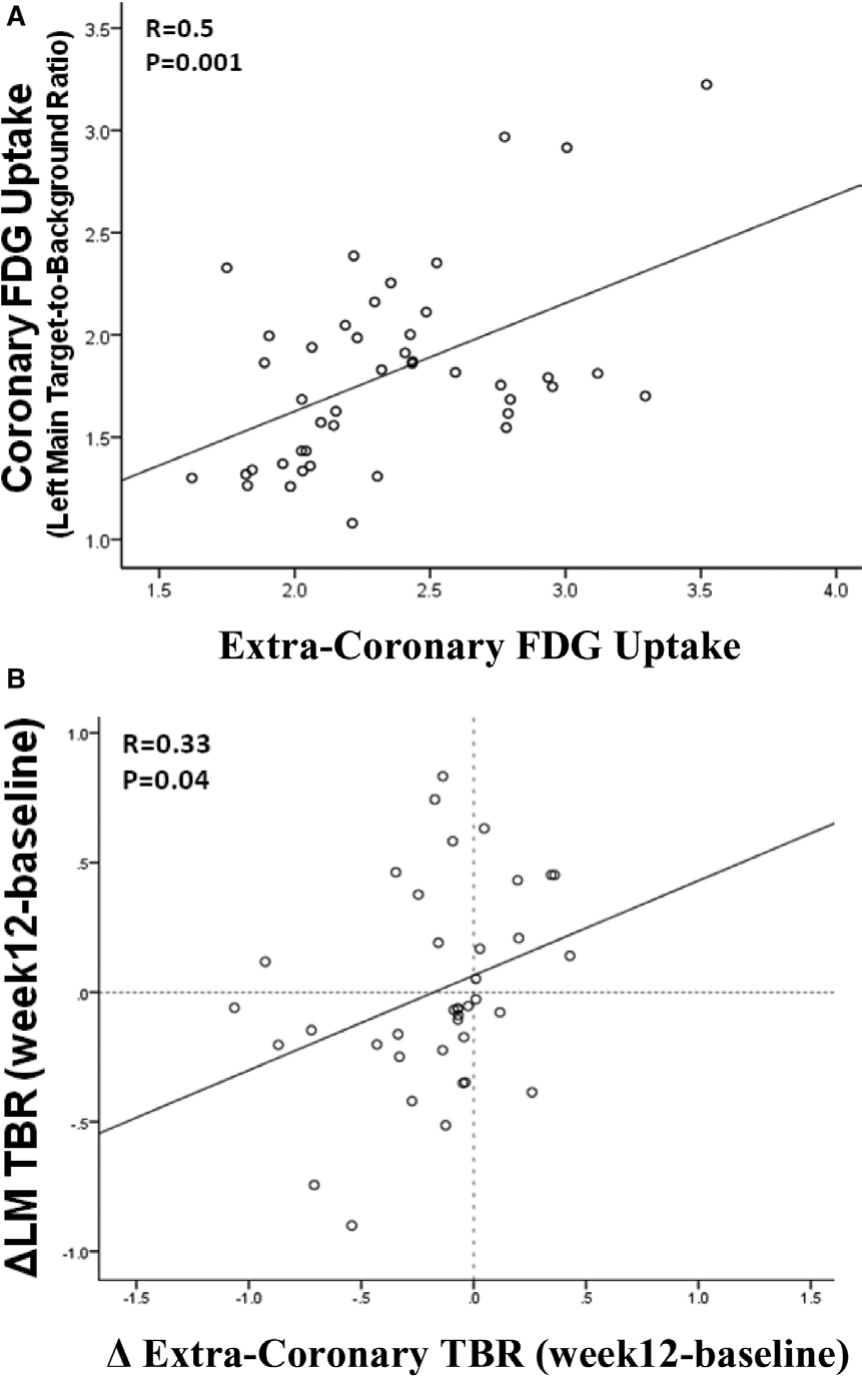
Extracoronary arterial ^18^F-flurodeoxyglucose (FDG) uptake parallels left main coronary artery (LMCA) FDG uptake. Index vessel FDG uptake (target-to-background ratio [TBR]) at baseline (**A**) and changes during the 12-wk treatment period (**B**) significantly correlated with baseline LMCA TBR and changes in LMCA TBR, respectively.

At baseline, LMCA inflammation (TBR) correlated with inflammation (TBR) in the aorta (*r*=0.6; *P*<0.001), carotid vessel (*r*=0.32; *P*=0.04), and index vessel (*r*=0.49; *P*=0.001; Figure 5A). Likewise, in response to 12-week atorvastatin treatment, changes in LMCA inflammation (TBR) correlated with changes in extracoronary arterial inflammation (ΔAorta TBR versus ΔLMCA TBR: *r*=0.51; *P*=0.001; ΔIndex vessel TBR versus ΔLMCA TBR: *r*=0.33; *P*=0.04; Figure 5B).

### Higher Extracoronary Arterial Inflammation Is Associated With Presence of High-Risk Coronary Structural Features

**Figure 6.**
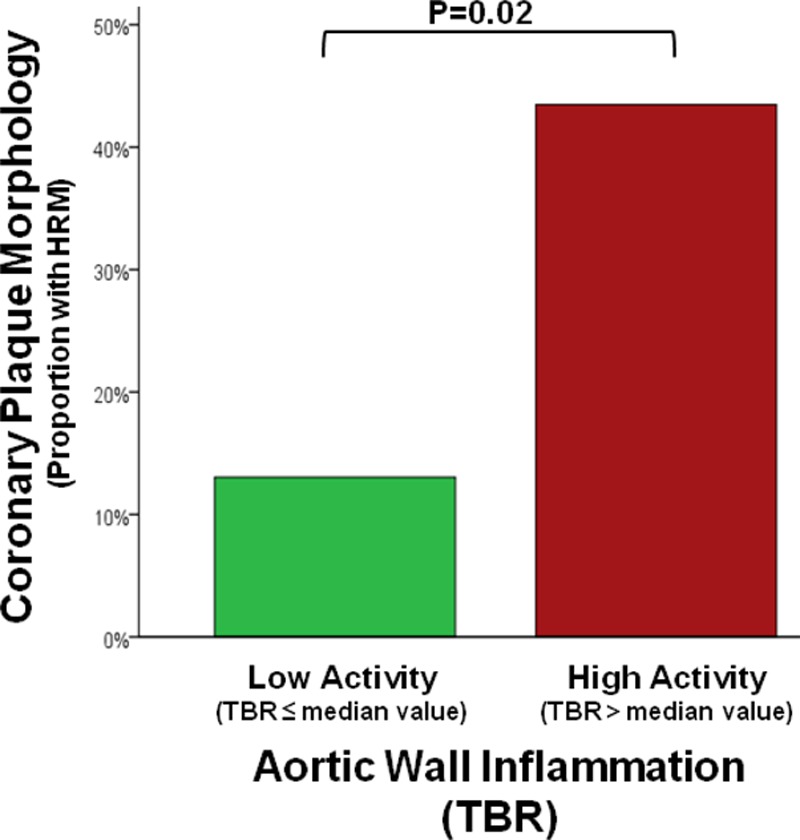
Extracoronary arterial inflammation associates with coronary structural features. The positron emission tomography (PET)/computed tomography (CT)–derived inflammatory signal in the ascending aorta (target-to-background ratio [TBR]) was associated with presence of high-risk coronary plaque features by computed tomography angiographic (CTA) such that subjects with higher aortic TBR (≥median) had increased frequency of high-risk plaque features (positive remodeling or low-attenuation plaque without dense calcification) in the entire coronary tree (43.5% vs 13%; *P*=0.02).

Furthermore, the TBR (inflammation) in the aorta was associated with the presence of HRM coronary plaque features (by CTA) such that subjects with higher aortic TBR (≥median) exhibited a higher frequency of high-risk structural plaque features throughout the coronary tree (44% versus 13%; *P*=0.02; Figure 6).

## Discussion

This first study examining the impact of statins on coronary artery inflammation yielded several novel observations. First, we provide initial evidence, in the coronary circulation, that coronary artery FDG accumulation, a marker of atherosclerotic inflammation, associates with HRM. Second, we demonstrate that statins result in a reduction in coronary inflammation, primarily in atherosclerotic plaques manifesting HRM. Furthermore, we demonstrate that statin-induced therapeutic modulation of extracoronary artery FDG uptake parallels changes in coronary arterial FDG uptake. Together, these findings highlight the systemic nature of atherosclerotic arterial inflammation and also suggest that statin therapy may have its greatest impact on more advanced atherosclerotic disease.

The key finding of this study is the observation that the anti-inflammatory effect of statins is seen primarily within more advanced lesions. The association between plaque morphology and statin effect was evident in both the coronary and extracoronary circulation and remained robust after correcting for potential confounders such as baseline TBR, prestudy statin use, low-density lipoprotein concentration, and statin dose. Accordingly, the findings of this study provide additional support for the hypothesis that assessment of atherosclerosis, including arterial wall inflammatory activity, might help to identify individuals who would derive the greatest benefit from statin therapy (even among individuals already deemed to be at increased risk using traditional risk calculators).^22,23^ This assertion is conceptually in-line with findings from the MESA study (Multi-Ethnic Study of Atherosclerosis), in which among individuals eligible for primary prevention, the majority of CHD and CVD events occurred in those with coronary artery calcium scores >100.^22^ From that, the authors suggested that preventative therapies should be preferentially prescribed to individuals with imaging evidence of more advanced atherosclerotic disease.

Similarly, Emami et al^24^ recently demonstrated that the presence of nonobstructive atherosclerotic plaques is associated with an increased risk of future atherosclerotic cardiovascular disease events and that incorporating information on nonobstructive coronary artery disease results in more accurate allocation of statin therapy. Our findings add an important additional consideration, by indicating that the underlying plaque morphology may additionally affect the local efficacy of statins. Together, these findings suggest that available imaging data might be useful for guiding treatment decisions on the use of statins or newer antiatherosclerotic approaches, especially in cases where there may be relative equipoise when deciding on primary prevention. Given the likely emergence of several new pharmacological tools (such as novel lipid-lowering drugs and anti-inflammatory drugs) with which to reduce the risk of incident atherothrombotic events, the medical community will likely experience a greater need to better allocate those new treatments, a paradigm that is in-line with the contemporary emphasis on precision medicine.^25^

In this study, we also tested the hypothesis that atherosclerotic inflammatory activity (represented as FDG uptake in LMCA) is highest in coronary plaques with HRM. Although the relationship between HRM and inflammation has been previously reported for the carotids,^26,27^ the current study is the first to extend that observation to the coronary circulation. This observation provides further validation of FDG-PET/CT imaging as a tool to characterize proximal coronary atherosclerosis, in that it demonstrates that coronary FDG uptake (by PET) associates with high-risk structural features that are identified using an orthogonal technique (CTA).

Furthermore, we investigated the relationship between inflammation in coronary and extracoronary vessels. In this evaluation, we observed several important associations. First, we found that individuals with higher extracoronary arterial inflammation had greater coronary inflammation and were also more likely to have coronary plaques containing high-risk structural features. Furthermore, we observed that the anti-inflammatory actions of statins were similarly manifested in the extracoronary and coronary circulation. These observations provide additional confidence that the data derived by looking at the large arteries (which are much more amenable to imaging) provide important insights into the coronary atherosclerotic milieu (where the atherothrombotic events of greatest concern originate). The data also provide additional insights as to why imaging of the aorta with FDG-PET/CT functions as an independent marker of risk of future CV events.^14,15^

Finally, we studied the relationship between both plaque morphology and inflammation and established serum proinflammatory biomarkers, MMP-3 and CRP. MMP-3 has been shown to correlate with inflammation (FDG uptake) in the aorta,^11^ and here, we replicate that finding but further demonstrate that this relationship extends to the coronary artery as well. Similar to other groups, we show a significant association between CRP and high-risk coronary plaque features.^28^ Collectively, our findings emphasize the systemic nature of atherosclerosis and arterial inflammation by providing insights on the biological links between resident plaques in both large and small arteries and circulating proinflammatory markers.

### Limitations

Of the patients enrolled in the larger randomized controlled prospective study, this study was limited to only those patients with evaluable CTA images and, thus, limits the generalizability of the results, In this subset of patients, a smaller proportion had CT evidence of high-risk plaques and perhaps may have limited our ability to detect a consistent relationship with CRP and MMP-3. It is also worth noting that analyses were not sufficiently powered to assess the impact of statin dose (high dose versus low dose) on LMCA.

In addition, coronary CTA was performed at a single time point, thereby precluding longitudinal assessment of changes or stability of coronary plaques at 12 weeks (after treatment). However, the reduction in both the coronary and extracoronary artery FDG signals, which has been validated histopathologically as an inflammatory marker,^12,26,29,30^ suggests that the underlying inflammation-mediated high-risk plaque features may have been favorably modified. Although the image analyses in our study were prespecified before initiation of any study procedures, future studies are warranted to confirm our findings.

### Conclusions

In summary, we show, for the first time, that statin therapy results in reduction in coronary inflammation and that the anti-inflammatory impact of statins is substantially greater within morphologically more advanced coronary plaques. Collectively, these findings suggest one potential mechanism by which statins may disproportionately benefit individuals with more advanced atherosclerotic disease.

## Sources of Funding

Dr Singh received support from the National Heart, Lung, and Blood Institute of the National Institutes of Health (5T32 HL076136) and Marfan Foundation. Dr Rudd is partly supported by the National Institute for Health Research Cambridge Biomedical Research Centre, the British Heart Foundation, and the Wellcome Trust.

## Disclosures

Drs Alon and Shankar are employees of Merck Sharp & Dohme Corp, a subsidiary of Merck & Co, Inc, Whitehouse Station, NJ. Dr Alon owns stock in Merck Sharp & Dohme Corp. Drs Tawakol and Fayad received consulting fees, and their institutions received grants from Merck Sharp & Dohme Corp. The other authors report no conflicts.

## CLINICAL PERSPECTIVE

Atherosclerotic disease is distributed inhomogeneously within the arterial wall. The presence of nonobstructive plaques, in particular, those with high-risk morphology, is associated with an increased risk of atherothrombosis. We tested the hypothesis that the anti-inflammatory effect of statins is greatest within left main coronary artery plaques containing high-risk morphology. Herein, we provide an initial observation that statin therapy results in a reduction in left main coronary artery inflammation. Furthermore, we show in both coronary and extracoronary arteries that the anti-inflammatory impact of statins is substantially greater within plaques that contain high-risk morphology. Together, the findings suggest an additional mechanism by which statins may disproportionately benefit individuals with more advanced atherosclerotic disease.

## References

[1] Motoyama S, Sarai M, Harigaya H, Anno H, Inoue K, Hara T, Naruse H, Ishii J, Hishida H, Wong ND, Virmani R, Kondo T, Ozaki Y, Narula J (2009). Computed tomographic angiography characteristics of atherosclerotic plaques subsequently resulting in acute coronary syndrome.. J Am Coll Cardiol.

[2] Motoyama S, Kondo T, Sarai M, Sugiura A, Harigaya H, Sato T, Inoue K, Okumura M, Ishii J, Anno H, Virmani R, Ozaki Y, Hishida H, Narula J (2007). Multislice computed tomographic characteristics of coronary lesions in acute coronary syndromes.. J Am Coll Cardiol.

[3] Pursnani A, Schlett CL, Mayrhofer T, Celeng C, Zakroysky P, Bamberg F, Nagurney JT, Truong QA, Hoffmann U (2015). Potential for coronary CT angiography to tailor medical therapy beyond preventive guideline-based recommendations: insights from the ROMICAT I trial.. J Cardiovasc Comput Tomogr.

[4] Blaha MJ, Budoff MJ, DeFilippis AP, Blankstein R, Rivera JJ, Agatston A, O’Leary DH, Lima J, Blumenthal RS, Nasir K (2011). Associations between C-reactive protein, coronary artery calcium, and cardiovascular events: implications for the JUPITER population from MESA, a population-based cohort study.. Lancet.

[5] Libby P (2002). Inflammation in atherosclerosis.. Nature.

[6] Cannon CP, Braunwald E, McCabe CH, Rader DJ, Rouleau JL, Belder R, Joyal SV, Hill KA, Pfeffer MA, Skene AM, Pravastatin or Atorvastatin Evaluation and Infection Therapy-Thrombolysis in Myocardial Infarction 22 Investigators (2004). Intensive versus moderate lipid lowering with statins after acute coronary syndromes.. N Engl J Med.

[7] Rudd JH, Myers KS, Bansilal S, Machac J, Pinto CA, Tong C, Rafique A, Hargeaves R, Farkouh M, Fuster V, Fayad ZA (2008). Atherosclerosis inflammation imaging with 18F-FDG PET: carotid, iliac, and femoral uptake reproducibility, quantification methods, and recommendations.. J Nucl Med.

[8] Rudd JH, Myers KS, Bansilal S, Machac J, Rafique A, Farkouh M, Fuster V, Fayad ZA (2007). (18)Fluorodeoxyglucose positron emission tomography imaging of atherosclerotic plaque inflammation is highly reproducible: implications for atherosclerosis therapy trials.. J Am Coll Cardiol.

[9] Tawakol A, Fayad ZA, Mogg R, Alon A, Klimas MT, Dansky H, Subramanian SS, Abdelbaky A, Rudd JH, Farkouh ME, Nunes IO, Beals CR, Shankar SS (2013). Intensification of statin therapy results in a rapid reduction in atherosclerotic inflammation: results of a multicenter fluorodeoxyglucose-positron emission tomography/computed tomography feasibility study.. J Am Coll Cardiol.

[10] Fayad ZA, Mani V, Woodward M, Kallend D, Abt M, Burgess T, Fuster V, Ballantyne CM, Stein EA, Tardif JC, Rudd JH, Farkouh ME, Tawakol A, dal-PLAQUE Investigators (2011). Safety and efficacy of dalcetrapib on atherosclerotic disease using novel non-invasive multimodality imaging (dal-PLAQUE): a randomised clinical trial.. Lancet.

[11] Rudd JH, Myers KS, Bansilal S, Machac J, Woodward M, Fuster V, Farkouh ME, Fayad ZA (2009). Relationships among regional arterial inflammation, calcification, risk factors, and biomarkers: a prospective fluorodeoxyglucose positron-emission tomography/computed tomography imaging study.. Circ Cardiovasc Imaging.

[12] Tawakol A, Migrino RQ, Hoffmann U, Abbara S, Houser S, Gewirtz H, Muller JE, Brady TJ, Fischman AJ (2005). Noninvasive *in vivo* measurement of vascular inflammation with F-18 fluorodeoxyglucose positron emission tomography.. J Nucl Cardiol.

[13] Pedersen SF, Graebe M, Fisker Hag AM, Højgaard L, Sillesen H, Kjaer A (2010). Gene expression and 18FDG uptake in atherosclerotic carotid plaques.. Nucl Med Commun.

[14] Rominger A, Saam T, Wolpers S, Cyran CC, Schmidt M, Foerster S, Nikolaou K, Reiser MF, Bartenstein P, Hacker M (2009). 18F-FDG PET/CT identifies patients at risk for future vascular events in an otherwise asymptomatic cohort with neoplastic disease.. J Nucl Med.

[15] Figueroa AL, Abdelbaky A, Truong QA, Corsini E, MacNabb MH, Lavender ZR, Lawler MA, Grinspoon SK, Brady TJ, Nasir K, Hoffmann U, Tawakol A (2013). Measurement of arterial activity on routine FDG PET/CT images improves prediction of risk of future CV events.. JACC Cardiovasc Imaging.

[16] Tahara N, Kai H, Ishibashi M, Nakaura H, Kaida H, Baba K, Hayabuchi N, Imaizumi T (2006). Simvastatin attenuates plaque inflammation: evaluation by fluorodeoxyglucose positron emission tomography.. J Am Coll Cardiol.

[17] Lehrer-Graiwer J, Singh P, Abdelbaky A, Vucic E, Korsgren M, Baruch A, Fredrickson J, van Bruggen N, Tang MT, Frendeus B, Rudd JH, Hsieh F, Ballantyne CM, Ghoshhajra B, Rosenson RS, Koren M, Roth EM, Duprez DA, Fayad ZA, Tawakol AA (2015). FDG-PET imaging for oxidized LDL in stable atherosclerotic disease: a phase II study of safety, tolerability, and anti-inflammatory activity.. JACC Cardiovasc Imaging.

[18] Rogers IS, Nasir K, Figueroa AL, Cury RC, Hoffmann U, Vermylen DA, Brady TJ, Tawakol A (2010). Feasibility of FDG imaging of the coronary arteries: comparison between acute coronary syndrome and stable angina.. JACC Cardiovasc Imaging.

[19] Nitta Y, Tahara N, Tahara A, Honda A, Kodama N, Mizoguchi M, Kaida H, Ishibashi M, Hayabuchi N, Ikeda H, Yamagishi S, Imaizumi T (2013). Pioglitazone decreases coronary artery inflammation in impaired glucose tolerance and diabetes mellitus: evaluation by FDG-PET/CT imaging.. JACC Cardiovasc Imaging.

[20] Nance JW, Schlett CL, Schoepf UJ, Oberoi S, Leisy HB, Barraza JM, Headden GF, Nikolaou K, Bamberg F (2012). Incremental prognostic value of different components of coronary atherosclerotic plaque at cardiac CT angiography beyond coronary calcification in patients with acute chest pain.. Radiology.

[21] Schlett CL, Ferencik M, Celeng C, Maurovich-Horvat P, Scheffel H, Stolzmann P, Do S, Kauczor HU, Alkadhi H, Bamberg F, Hoffmann U (2013). How to assess non-calcified plaque in CT angiography: delineation methods affect diagnostic accuracy of low-attenuation plaque by CT for lipid-core plaque in histology.. Eur Heart J Cardiovasc Imaging.

[22] Martin SS, Blaha MJ, Blankstein R, Agatston A, Rivera JJ, Virani SS, Ouyang P, Jones SR, Blumenthal RS, Budoff MJ, Nasir K (2014). Dyslipidemia, coronary artery calcium, and incident atherosclerotic cardiovascular disease: implications for statin therapy from the multi-ethnic study of atherosclerosis.. Circulation.

[23] Bittencourt MS, Blaha MJ, Blankstein R, Budoff M, Vargas JD, Blumenthal RS, Agatston AS, Nasir K (2014). Polypill therapy, subclinical atherosclerosis, and cardiovascular events-implications for the use of preventive pharmacotherapy: MESA (Multi-Ethnic Study of Atherosclerosis).. J Am Coll Cardiol.

[24] Emami H, Singh P, MacNabb M, Vucic E, Lavender Z, Rudd JH, Fayad ZA, Lehrer-Graiwer J, Korsgren M, Figueroa AL, Fredrickson J, Rubin B, Hoffmann U, Truong QA, Min JK, Baruch A, Nasir K, Nahrendorf M, Tawakol A (2015). Splenic metabolic activity predicts risk of future cardiovascular events: demonstration of a cardiosplenic axis in humans.. JACC Cardiovasc Imaging.

[25] Collins FS, Varmus H (2015). A new initiative on precision medicine.. N Engl J Med.

[26] Figueroa AL, Subramanian SS, Cury RC, Truong QA, Gardecki JA, Tearney GJ, Hoffmann U, Brady TJ, Tawakol A (2012). Distribution of inflammation within carotid atherosclerotic plaques with high-risk morphological features: a comparison between positron emission tomography activity, plaque morphology, and histopathology.. Circ Cardiovasc Imaging.

[27] Graebe M, Pedersen SF, Højgaard L, Kjaer A, Sillesen H (2010). 18FDG PET and ultrasound echolucency in carotid artery plaques.. JACC Cardiovasc Imaging.

[28] Rubin J, Chang HJ, Nasir K, Blumenthal RS, Blaha MJ, Choi EK, Chang SA, Yoon YE, Chun EJ, Choi SI, Agatston AS, Rivera JJ (2011). Association between high-sensitivity C-reactive protein and coronary plaque subtypes assessed by 64-slice coronary computed tomography angiography in an asymptomatic population.. Circ Cardiovasc Imaging.

[29] Menezes LJ, Kotze CW, Agu O, Richards T, Brookes J, Goh VJ, Rodriguez-Justo M, Endozo R, Harvey R, Yusuf SW, Ell PJ, Groves AM (2011). Investigating vulnerable atheroma using combined (18)F-FDG PET/CT angiography of carotid plaque with immunohistochemical validation.. J Nucl Med.

[30] Tawakol A, Migrino RQ, Bashian GG, Bedri S, Vermylen D, Cury RC, Yates D, LaMuraglia GM, Furie K, Houser S, Gewirtz H, Muller JE, Brady TJ, Fischman AJ (2006). *In vivo* 18F-fluorodeoxyglucose positron emission tomography imaging provides a noninvasive measure of carotid plaque inflammation in patients.. J Am Coll Cardiol.

